# Points

**Published:** 1898-11

**Authors:** 


### POINTS.

North Carolina will ask from members of the veterinary and medical profession, laws to better regulate animal-food inspection at the coming session of the State Legislature.

Philadelphia veterinarians will move this winter on the dispensers of milk whose wagons and places of business are emblazoned with letters claiming veterinary and sanitary inspection of dairies, many of whom make only the merest pretence to the proper performance of this work.

Under a more thorough inspection tuberculous carcasses have increased from 7 to 25 per cent. in Saxony's slaughter-houses.

The Maine Veterinary Medical Association will introduce a bill at the next session of the Legislature looking to the establishing of a State examining and registration board.

